# Correlation between TCM Syndromes and Type 2 Diabetic Comorbidities Based on Fully Connected Neural Network Prediction Model

**DOI:** 10.1155/2021/6095476

**Published:** 2021-11-20

**Authors:** Yifei Wang, Runshun Zhang, Min Pi, Julia Xu, Moyan Qiu, Tiancai Wen

**Affiliations:** ^1^Wangjing Hospital, China Academy of Chinese Medical Sciences, Beijing, China; ^2^Guang'anmen Hospital, China Academy of Chinese Medical Sciences, Beijing, China; ^3^Shenzhen Traditional Chinese Medicine Hospital, Shenzhen, Guangdong, China; ^4^The University of Melbourne, Melbourne, Australia; ^5^Data Center of Traditional Chinese Medicine, China Academy of Chinese Medical Sciences, Beijing, China; ^6^Institute of Basic Research in Clinical Medicine, China Academy of Chinese Medical Sciences, Beijing, China

## Abstract

**Objective:**

To predict the major comorbidities of type 2 diabetes based on the distribution characteristics of syndromes, and to explore the relationship between TCM syndromes and comorbidities of type 2 diabetes.

**Methods:**

Based on the electronic medical record data of 3413 outpatient visits from 995 type 2 diabetes patients with comorbidities, descriptive statistical methods were used to analyze the basic characteristics of the population, the distribution characteristics of comorbidities, and TCM syndromes. A neural network model for the prediction of type 2 diabetic comorbidities based on TCM syndromes was constructed.

**Results:**

Patients with TCM syndrome of *blood amassment in the lower jiao* were diagnosed with renal insufficiency with 95% test sensitivity. The patients with *spleen deficiency* combined with *ascending counterflow of stomach qi* and *cold-damp* patterns were diagnosed with gastrointestinal lesions with 92% sensitivity. The patients with TCM syndrome group of *spleen heat* and *exuberance of heart fire* were diagnosed as type 2 diabetes complicated with hypertension with a sensitivity of 91%. In addition, the prediction accuracy of combined neuropathy, heart disease, liver disease, and lipid metabolism disorder reached 70∼90% in TCM syndrome groups.

**Conclusion:**

The fully connected neural network model study showed that syndrome characteristics are highly correlated with type 2 diabetes comorbidities. Syndrome location is commonly in the heart, spleen, stomach, lower *jiao*, meridians, etc., while syndrome pattern manifests in states of *deficiency*, *heat*, *phlegm,* and *blood stasis*. The different combinations of disease location and disease pattern reflect the syndrome characteristics of different comorbidities forming the characteristic syndrome group of each comorbidity. Major comorbidities could be predicted with a high degree of accuracy through TCM syndromes. Findings from this study may have further implementations to assist with the diagnosis, treatment, and prevention of diabetic comorbidities at an early stage.

## 1. Introduction

Type 2 diabetes mellitus (T2DM) is a chronic and progressive disease with multiple etiologies over a long course. It seriously affects the quality of life of patients and increases the risk of early death [1]. IDF estimated that approximately 4.2 million adults will die as a result of diabetes and its complications in 2019. Globally, 11.3% of deaths are due to diabetes. Almost half of these deaths are in people under 60 years of age [2]. Studies have found that more than 40% of diabetics suffer from comorbidities [3]. Comorbidity is an important factor affecting the prognosis of diabetics, and some comorbidities are also major causes of disability and death.

Type 2 diabetes develops mainly due to insulin resistance and relative insulin insufficiency and usually manifests as a glycolipid metabolism disorder [4, 5]. As diabetes progresses over time, various comorbidities may develop. In recent years, many studies have shown that complementary and alternative medicine therapies including traditional Chinese medicine (TCM) have good efficacy in improving insulin resistance and regulating glycolipid metabolism with a high degree of safety [5–8]. At the same time, the application of relevant complementary alternative medicine therapies is of great significance in improving the quality of life of diabetic patients and preventing the occurrence and development of diabetes-related comorbidities. However, long-term hyperglycemia in diabetics affects multiple organs over time. Therefore, in the treatment of diabetes with complementary and alternative medicine, we must first accurately diagnose diabetes mellitus according to a theoretical system.

TCM is a common and important model of complementary and alternative medicine. It is the crystallization of thousands of years of medical experience and wisdom of the Chinese nation [9]. TCM has unique insights into the diagnosis and treatment of diabetes and its comorbidities and has been shown to effectively control the progression of diabetes, as well as diagnose, prevent, and treat diabetes mellitus at an early stage. TCM emphasizes the concepts of “holism” and “syndrome differentiation and treatment”. “Syndrome differentiation” is the essence of TCM diagnosis and treatment of disease. TCM views that disease symptoms reflect the nature of a particular internal and external environment at a certain stage of the disease or the individual patient at the time. It manifests in corresponding patterns observed in the tongue, pulse, shape, color, and complexion. Patterns can also reveal aspects of pathogenesis such as etiology, disease location, disease nature, and disease status to various degrees, providing a basis for syndrome differentiation and treatment [10]. Different diseases contain different progression patterns of syndromes [11]. Syndromes usually reflect the current state of the disease. Being able to clarify the relationship between syndromes and various comorbidities will assist with the diagnosis of type 2 diabetes comorbidities based on syndrome characteristics, which is important for early treatment and prevention. Based on real clinical medical records, this study analyzed the relationship between TCM syndromes and comorbidities. A prediction model of “TCM syndrome-comorbidity” based on a neural network was also constructed to provide a reference for the diagnosis of diabetes comorbidity.

## 2. Data and Methods

### 2.1. Data Sources

The data for this study was obtained from the electronic medical records of T2DM patients, which were collected in the database of the Data Center of Traditional Chinese Medicine, China Academy of Chinese Medical Sciences. Data screening rules were as follows: (1) the primary diagnosis being type 2 diabetes, (2) at least one secondary diagnosis, (3) age ≥18 years, and (4) complete information about TCM and Western medicine diagnosis and TCM syndrome diagnosis. To protect privacy, all personal information such as patient names and phone numbers was desensitized prior to the study, and medical record numbers were used to identify each patient. Data regarding patient ID, gender, age, frequency of visit, course of the disease, disease diagnosis, and syndrome diagnosis was extracted. This study was approved by the Ethics Committee of the Institute of Basic Research in Clinical Medicine, China Academy of Chinese Medical Sciences (approval number:2016 No.11-01).

### 2.2. Data Standardization and Classification

In cases where disease names were not standardized (terms have different semantic expressions), medical professionals matched the original data with the standard disease names in the *International Classification of Diseases 10th edition* (ICD-10) [12]. For example, cerebral infarction (lacunar) was standardized as lacunar infarction. Next, the standardized disease names were classified for statistical analysis. For example, bile duct stones, bile duct dilatation, and cholangitis were categorized into bile duct disease.

There were problems with the standardization of the TCM syndrome diagnosis such as classification confusion and different names of the same syndrome. In these circumstances, the compound syndrome was divided into two or several single syndromes and standardized according to *Clinic terminology of traditional Chinese medical diagnosis and treatment • Syndromes (GB/T16751.2- 1997)* [13] and *syndrome element syndrome differentiation (SESD)* [14]. For example, the “*qi and yin deficiency with blood stasis*” syndrome was divided into *qi deficiency*, *yin deficiency,* and *blood stasis*.

### 2.3. Data Analysis

#### 2.3.1. Descriptive Analysis

Microsoft Excel 2019 was used to count the gender, age, distribution of visits, and frequency of comorbidities of all patients in the included case data. Continuous variables with normal distribution were represented by mean ± standard deviation (SD) and the continuous variables with nonnormal distribution were represented by median and interquartile ranges (M (Q1, Q3)). Categorical variables were described by frequency and percentage (n (%)).

#### 2.3.2. Construction of Neural Network Model

In this study, Python 3.6 was used to construct a fully connected neural prediction model from TCM syndromes to comorbidities. A fully connected neural network is the oldest data mining model, which has the most network parameters and the largest amount of computation. It consists of an input layer, hidden layer, and output layer, and the “neurons” in each layer are interconnected with the neurons in the next layer in a fully connected mapping model [15]. The input layer is the independent variable. The output layer is the dependent variable. The hidden layer and the number of nodes determine the complexity of the network model. The neural network first randomly assigns weights to the independent variables and then compares the predicted results with the known results. The prediction error of the hidden nodes in the upper layer is estimated by the prediction error of the output layer, and the error is back-propagated to the input layer from back to forward layer by layer, so as to adjust the link weight and find the best diagnostic model.

**Figure 1:**
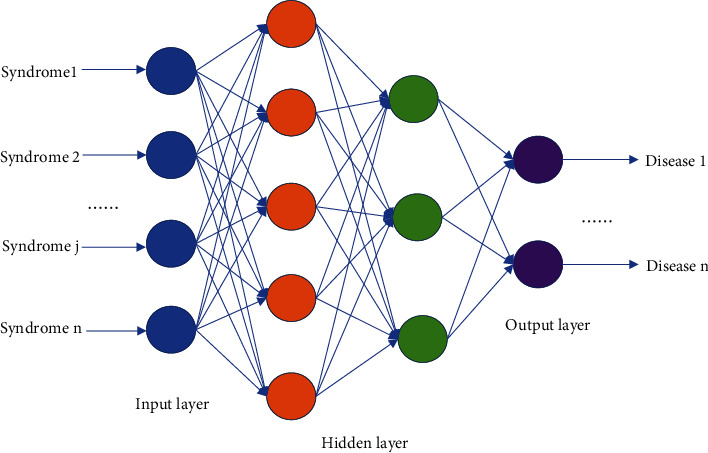
Neural network model of predicted TCM syndromes and comorbidities.

In this study, a four-layer fully connected neural network model was adopted. Descriptive analysis results were used to select representative syndromes and comorbidities. Label encoding of TCM syndrome diagnosis information was taken as the input layer and main diabetic comorbidities as the output layer, and the hidden layer was set as 2 layers (Figure 1).

**Figure 2:**
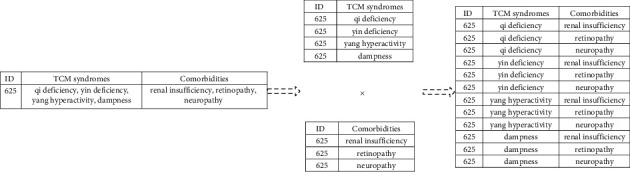
Example of “syndrome-comorbidity” construction process.

In order to adapt to the neural network model, the relationship data of “multiple syndromes-multiple comorbidities” in the original data was split to form the data tables of “single syndrome” and “single comorbidity” in this study. The two tables were then fully joined to form the “syndrome- comorbidity” data table (Figure 2). The single syndrome corresponds to the neural network's input layer, and the single comorbidity corresponds to the neural network's output layer.

When the neural network model was trained, more than 80% of samples were randomly selected from the “syndrome-comorbidity” data set as the training set, and the rest of the data selected as the test set. The training was considered complete when the calculated results of the model loss function became relatively stable. Finally, a neural network model for predicting the comorbidities of diabetes was established using TCM syndrome patterns. The calculation results of the final model in the prediction set were taken out to calculate the predictive probability of different syndrome groups for comorbidities.

## 3. Results

### 3.1. General Information

A total of 3413 medical records of 995 T2DM patients were included in this study, which comprised 158 types of diabetic comorbidities and 188 types of TCM syndromes. Of all patients, 470 were male (47.24%) and 525 were female (52.76%). In terms of age, the patients were mainly distributed in the range of 40 to 80 years old, including 465 (46.73%) aged 40–59 years and 374 (37.59%) aged 60–79 years. The number of visits ranged from 1 to 36, with a median of 2 (1–4) visits, and the number of patients with 1–3 visits was the largest. Patients were mainly complicated with 1-2 diseases and were mostly associated with 1-2 syndromes (Table 1).

**Figure 3:**
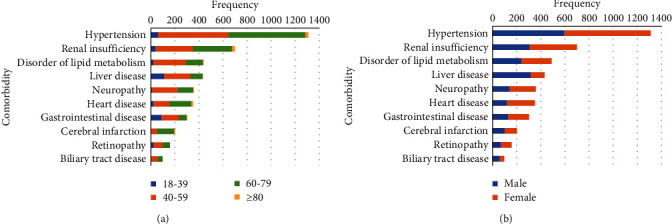
Distribution of comorbidities. (a) Age. (b) Gender.

**Figure 4:**
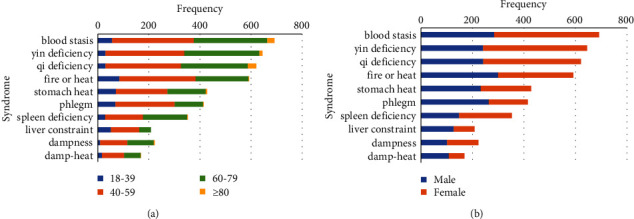
Distribution of syndromes. (a) Age. (b) Gender.

The top 10 comorbidities included hypertension, renal insufficiency, disorder of lipid metabolism, liver disease, neuropathy, heart disease, gastrointestinal disease, cerebral infarction, retinopathy, and biliary tract disease, accounting for 82.36% of all comorbidities (Figure 3). Compared with other age groups, there were more patients with liver diseases and gastrointestinal diseases in the 18–39 age group, and the number of patients with cardiovascular and cerebrovascular diseases increased significantly with age (Figure 3(a)). In terms of gender distribution, there were far more males than females in patients with liver disease (Figure 3(b)). The most common TCM syndromes included *blood stasis*, *yin deficiency, qi deficiency*, *fire or heat*, *stomach heat*, *phlegm*, spleen deficiency, liver constraint, dampness, and damp-heat (Figure 4). The syndrome manifestations of patients aged 18–39 years were mainly excess syndromes, such as blood stasis, fire or heat, stomach heat, phlegm, and liver constraint. The elderly over 80 years old showed obvious qi deficiency, yin deficiency, and blood stasis (Figure 4(a)). Female patients with qi deficiency, yin deficiency, and blood stasis were slightly higher in number than males with those symptoms (Figure 4(b)).

### 3.2. Syndrome Characteristics of Different Comorbidities

**Figure 5:**
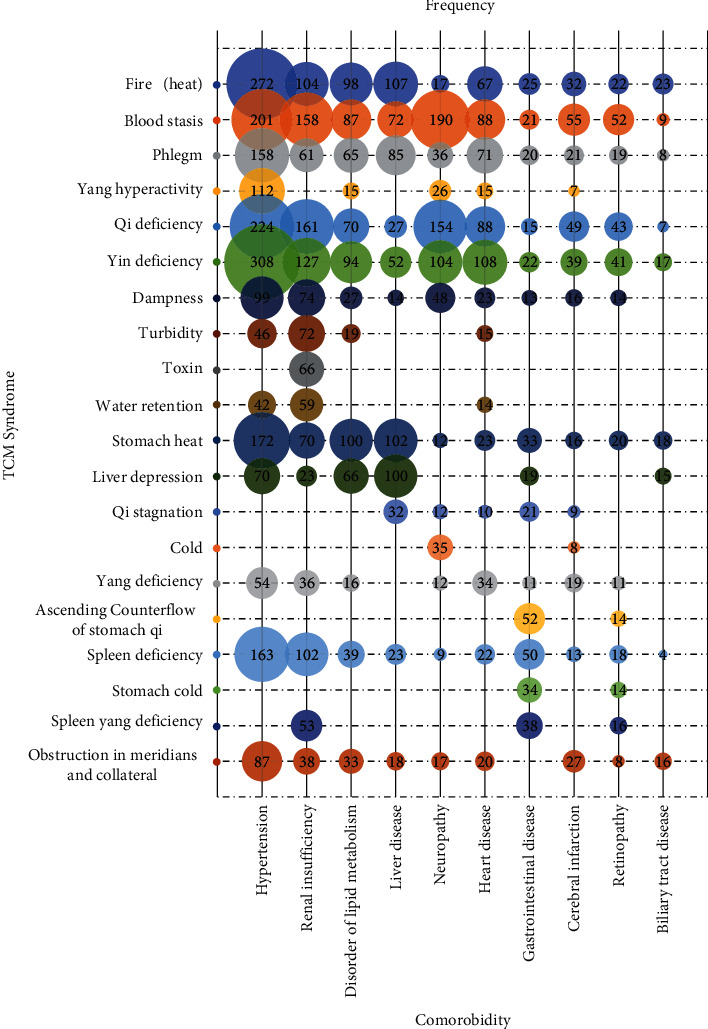
Syndrome distribution of comorbidities.

This study conducted a statistical analysis on the syndrome distribution characteristics of the top 10 comorbidities (Figure 5). It was found that there were obvious differences in the distribution characteristics, location, and nature of syndromes of different diseases. For example, in patients with diabetes mellitus complicated with hypertension, patterns of *deficiency* and *excess* were present in their syndrome manifestations. *Deficiency syndromes* were mainly *yin deficiency* and *qi deficiency*. The *excess syndromes* were *fire or heat*, *blood stasis*, *phlegm,* and *yang hyperactivity*. The syndromes of patients with renal insufficiency were mainly *qi deficiency*, *blood stasis*, *yin deficiency,* and *fire (heat)*. At the same time, the syndrome characteristics of *turbidity*, *toxin*, *dampness,* and *water retention* were highlighted. Patients with liver disease mostly had liver and stomach organ lesions. The main syndrome characteristic was the disorder of *qi* movement in the middle *jiao*, and the syndrome manifestations included *stomach heat*, *liver constraint*, *fire or heat*, *phlegm*, and *qi stagnation*. Patients with neuropathy mainly showed *deficiency syndrome* and *stasis syndrome*, including *qi deficiency*, *blood stasis*, *yin deficiency*, and *coldness*. The disease location of combined heart disease was mainly in the heart, and the main syndromes included *qi deficiency*, *yin deficiency*, *blood stasis*, and *yang deficiency*.

### 3.3. Evaluation of “Syndrome-Comorbidity” Neural Network Model

**Figure 6:**
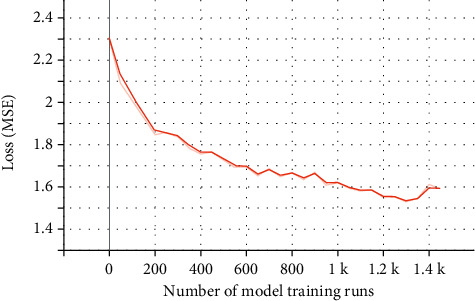
Evaluation of predictive neural network of TCM syndromes and comorbidities in diabetic patients.

There was a total of 4375 “syndrome-comorbidity” data cases for diabetes, with 188 syndrome diagnoses, which formed a 4375 × 188 label-encoding characteristic matrix. The top 10 comorbidities of diabetes were selected as the output label Y, forming a 4375 × 10 Label-encoding matrix. The original data was divided into a training set and a test set. 3875 cases of data were randomly selected from the feature matrix X as the training set and the remaining data as the test set. After repeated training of the model, convergence was reached at 1400 runs (Figure 6).

MSE is the mean square error, which is the average value of the square of the distance between the predicted value *f*(*x*) of the model and the real value *y* of the sample. It is a type of loss function which is used to evaluate the degree of inconsistency between the predicted value *f*(*x*) of the model and the real value *y*.

### 3.4. “Syndrome-Disease” Predictive Relationships for Major Comorbidities

**Figure 7:**
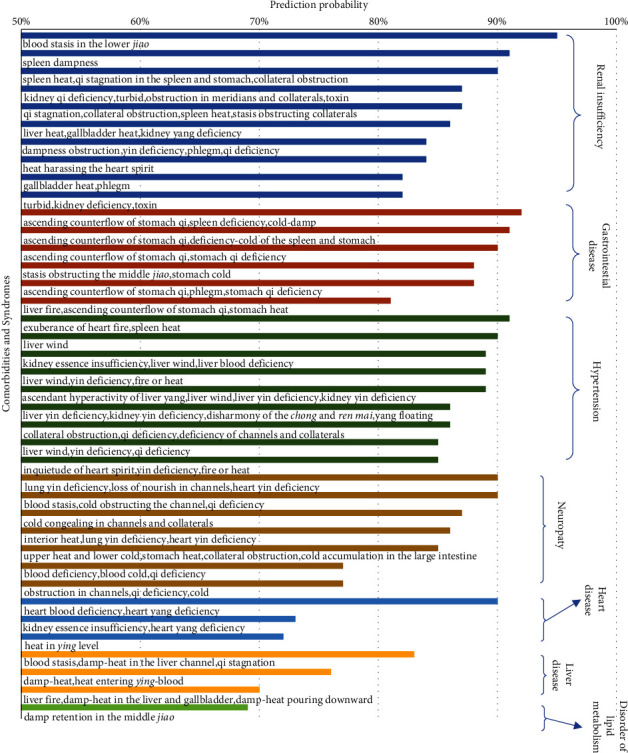
Probability of predicting comorbidity by different syndrome groups.

Through detailed analysis of the prediction results of the model, it was found that the accuracy of the model in predicting the seven comorbidities of renal insufficiency, gastrointestinal disease, hypertension, neuropathy, heart disease, liver disease, and lipid metabolism disorder could reach 70∼90%, while the prediction accuracy for cerebral infarction was about 50%, and the prediction accuracy for retinopathy and biliary tract disease was below 50% (Figure 7). The results showed that different characteristic syndrome groups could be used to predict the corresponding comorbidities.

Renal insufficiency was the most predictive comorbidity. Patients with TCM syndrome of *blood amassment in the lower jiao* were diagnosed with renal insufficiency with 95% accuracy. Patients with syndrome groups of *kidney qi deficiency, turbid, toxic,* and *obstruction in meridians and collaterals* were diagnosed with renal insufficiency with a sensitivity of 87%. It could also be seen from other TCM syndrome groups that the syndrome was dominated by *interior patterns*, and patients with *turbidity*, *toxin*, *stasis,* and *deficiency* of the kidney and spleen had a higher probability of being diagnosed with combined renal insufficiency.

The gastrointestinal disease was also comorbidity with characteristic syndrome groups and high accuracy of prediction. Patients with *spleen deficiency* combined with *ascending counterflow of stomach qi* and *cold-damp* patterns were diagnosed with gastrointestinal lesions with 92% sensitivity. The syndrome group of *ascending counterflow of stomach qi* and *deficiency-coldness of the spleen and stomach* were diagnosed with diabetes combined with the gastrointestinal disease with 91% sensitivity. It could also be observed from the other TCM syndrome groups that the main locations of disease were in the stomach, spleen, and middle *jiao*, and patients with the syndrome groups of *deficiency*, *qi counterflow*, *coldness,* and *blood stasis* had a high probability of being diagnosed with diabetes complicated with gastrointestinal disease.

For patients with hypertension, the probability of the syndrome group predicting the comorbidity was also relatively high. 92% of patients with TCM syndrome group of *spleen heat* and *exuberance of heart fire* were diagnosed with type 2 diabetes complicated with hypertension. 89% of patients with the syndrome group of *liver wind*, *ascendant hyperactivity of liver yang*, *liver yin deficiency,* and *kidney yin deficiency* were diagnosed with diabetes combined with hypertension. 86% of patients with TCM syndrome group of *liver yin deficiency*, *kidney yin deficiency*, *disharmony of the chong and ren mai,* and *yang floating* were diagnosed with hypertension. It could also be seen from the other TCM syndrome groups that a portion of patients with liver or kidney as the main location of disease and *deficiency* or *wind* as the main nature of disease were diagnosed as diabetes complicated with hypertension.

The results of this study showed that different syndrome groups could predict corresponding comorbidities. The syndromes of *fire or heat*, *qi stagnation*, *blood stasis,* and *damp-heat* were associated with the diagnosis of diabetes complicated with liver disease. A high portion of patients who had a location of disease mainly in the heart and *kidney,* nature of the disease being *deficiency* and *coldness,* were diagnosed with diabetes complicated with heart disease. Those with *blood stasis*, *coldness,* and *deficiency* as the main syndrome manifestations were diagnosed with type 2 diabetes complicated with neuropathy with high sensitivity. *Damp retention in the middle jiao* was the characteristic symptom in the diagnosis of type 2 diabetes mellitus combined with lipid metabolism disorder. However, among all TCM syndrome groups, the probability of being diagnosed with diabetes combined with cerebral infarction, retinopathy, and biliary tract disease was relatively low, and no syndrome group with a prediction rate of more than 60% was found. 59% of those with TCM syndrome of *kidney essence deficiency* and *cerebrospinal damage* were diagnosed with cerebral infarction, and 56% of patients with the independent syndrome of *wind* were diagnosed with diabetes mellitus combined with cerebral infarction.

## 4. Discussion

### 4.1. Distribution Characteristics and Overall Relationship between Diabetic Comorbidities and TCM Syndromes

The results of this study showed that the common syndromes of diabetic patients with comorbidity were a combination of *deficiency* and *excess*. *Excess syndrome* mainly consists of *heat syndrome*, *blood stasis syndrome,* and *phlegm syndrome*. *Qi deficiency* and *yin deficiency* were the main patterns of the *deficiency syndrome*. The number of younger diabetic patients with digestive system diseases was higher in comparison to the elderly, who were more likely to be complicated with cardiovascular and cerebrovascular diseases. In terms of syndrome manifestation, younger patients showed a higher incidence of the *excess syndrome*, while the elderly patients showed a higher incidence of *deficiency syndrome* and *blood stasis*. In terms of gender distribution, male patients were more likely to be complicated with liver diseases, while patients with *qi deficiency*, *yin deficiency,* and *blood stasis syndrome* were mostly female. According to TCM, diabetes is a chronic progressive disease, and the pathogenesis is a *deficiency-excess* complex. The core pathogenesis of diabetes is “*deficiency*, *heat,* and *blood stasis*” [16, 17]. At the same time, *phlegm* is also an important pathological factor in diabetes mellitus [18]. This is consistent with the results of this study. In addition, *constraint-heat* is the main pathogenesis in the early stage of diabetes and gradually progresses to the stage of *deficiency* and *detriment* with the development of the disease [19]. The influence of age on the distribution of diseases and syndromes is related to the modern living and working environment, physique, course of the disease, and other factors. Gender distribution is a dividing factor that may relate to physiology and living environment differences.

The results of this study showed that there were obvious differences in the syndrome distribution characteristics of different comorbidities, and the prediction result of the neural network showed that the prediction of comorbidities by characteristic syndrome group had high accuracy. Studies have shown that untreated type 2 diabetes is prone to various acute or chronic complications when the body is in a high glucose state for a prolonged period of time [20], and with the progression of the disease, the distribution characteristics and symptoms change accordingly [21]. This shows that there is a close correlation between different comorbidities and TCM syndromes. Different pathogenesis often leads to the occurrence of different comorbidities, the characteristics of the comorbidities are significant in the manifestations of TCM syndromes, and there will be some syndrome groups with very obvious characteristics in different diabetic comorbidities. Characteristic syndrome groups can be an important factor in distinguishing the corresponding comorbidities.

### 4.2. Syndrome Characteristics of Different Comorbidities

#### 4.2.1. Hypertension

The results of this study showed that hypertension was the most common comorbidity. Its TCM syndromes were characterized by *liver wind*, *fire or heat*, *yin deficiency,* and *disharmony of the chong and ren mai*, and the location of the disease was mainly reflected in the kidney and liver. Patients with TCM syndrome group of *spleen heat* and *exuberance of heart fire* were diagnosed as type 2 diabetes complicated with hypertension with a sensitivity of 91%. TCM considers “*liver-kidney yin deficiency*” as the root (the mean) and the “*wind*, *fire*, *phlegm,* and *blood stasis*” as the branch (the secondary) in terms of hypertension pathogenesis [22–24]. Dating back to as early as in *The Yellow Emperor's Inner Classic: Basic Questions*, there was a description of the pathogenesis of hypertensive vertigo: “*All wind with vertigo is ascribed to the liver*”. Meanwhile, *disharmony of the chong and ren mai* is also a common symptom of hypertension in perimenopausal women [25, 26]. With the increase of blood glucose, diabetes patients usually suffer from hunger, weight loss, constipation, frequent urination, and other symptoms, which is the main manifestation of “*Stagnancy of Er Yang*”, just like what is described in *The Yellow Emperor's Inner Classic:* “*Disorder of Stomach may be Affected by Disorders of the Heart or Spleen*” [27]. *Exuberant heat* in the heart and spleen combined with emotional depression leads to the *binding constraint of liver qi* and induces *ascendant hyperactivity of liver yang* and results in the occurrence of hypertension. The prediction results of hypertension in this study are consistent with Traditional Chinese medicine theory and clinical practice.

#### 4.2.2. Renal Insufficiency

Renal insufficiency is also common comorbidity of diabetes. The results of this study showed that in addition to the syndrome of “*blood stasis*, *heat,* and *deficiency*”, the syndromes of *turbidity*, *poison,* and *dampness* were also associated with renal insufficiency. At the same time, patients with TCM syndrome of *blood amassment in the lower jiao* were diagnosed with renal insufficiency with 95% sensitivity. Those with the syndrome group of *kidney qi deficiency*, *turbid*, *toxic*, and *obstruction in meridians and collaterals* were diagnosed with diabetes complicated with renal insufficiency with a sensitivity of 87%. The kidney is located in the lower *jiao*. With further development of the disease, kidney essence is damaged, causing the failure in *qi* transformation, which creates difficulty in distinguishing the clear and metabolic turbidity, leading to the *accumulation of turbid pathogens* and *water-dampness* and formation of *turbid-toxin*. *Turbid-toxin* obstruction in the collateral and consequent unsmooth blood circulation results in *water retention* which then leads to edema and eventually results in kidney disease [28]. Meanwhile, *turbidity-toxin* is closely related to glucose toxicity and lipid toxicity in diabetes mellitus [29]. *Removing turbidity and resolving toxins* are also important priorities in treating diabetic nephropathy [30–32]. The occurrence of related diseases can be actively prevented according to the symptoms, which is also of great significance in controlling the progression of diabetes.

#### 4.2.3. Neuropathy

The characteristic syndromes of diabetes complicated with neuropathy were *malnutrition of channels* and *coldness coagulation in the meridians and collaterals*. The syndrome group of “*blood stasis*, *qi deficiency*, and *cold coagulation in meridians and collaterals*” could predict diabetic neuropathy with an accuracy of 90%. In the early stage of diabetes, “*heat syndrome*” is the main syndrome, whereby the pathogenic *heat* damages the essential *qi* of zang-fu organs, especially the spleen. *Spleen yang deficiency* cannot reach the end of the limbs, resulting in a counterflow coldness in the four limbs. The *coldness congealment* leads to *blood stasis*, which results in *blood bi* and *coldness coagulation in meridians and collaterals* [33]. This is also consistent with the understanding of diabetes complicated with neuropathy in TCM. It is believed that the location of this disease is mainly in the channels and collaterals, involving the liver, kidney, and spleen. *Qi and blood deficiency* are considered as the root of pathogenesis and the *blood stasis obstructing collaterals* as the branch [34].

#### 4.2.4. Other Comorbidities

In addition, there was also a close relationship between TCM syndromes and other comorbidities. Different comorbidities have their own syndrome groups with obvious characteristics. For example, the syndrome characteristics of liver diseases are *damp-heat*, *qi stagnation,* and *blood stasis*. *The binding constraint of liver qi* and *liver-gallbladder damp-heat* are two new syndromes that have evolved in modern life, while *stasis syndrome* is often the key to disability and life-threatening diabetic complications [35]. The syndrome characteristics of combined heart disease are mainly *heart qi deficiency* or *heart yang deficiency*. The characteristic syndromes of patients complicated with gastrointestinal disease were *ascending counterflow of stomach qi*, *stomach qi deficiency,* and *stomach coldness*. The syndrome of disorder of lipid metabolism is characterized by *dampness obstruction in the middle jiao*. It can be seen that with the progression of diabetes, the TCM syndromes also evolve correspondingly, and the TCM syndromes show different manifestations during the occurrence of different comorbidities, indicating that the comorbidities are closely related to the TCM syndromes. It is of great practical significance to be able to predict the associated comorbidities through characteristic syndromes in the diagnosis, treatment, and prevention of diabetes mellitus.

## 5. Conclusion

In summary, this paper introduced a fully connected neural network approach to the study of the association between type 2 diabetic comorbidities and TCM symptoms with the construction of a “TCM symptom-comorbidity” prediction model. Different kinds of comorbidities were characterized by “*deficiency*, *heat*, *phlegm*, and *blood stasis*” in the viscera of diabetic patients, such as the heart, spleen, stomach, and kidney. According to the characteristics of the disease, different syndrome elements were combined to form characteristic syndrome groups. The corresponding comorbidities of diabetes were predicted based on the characteristics of the syndrome groups with a high degree of accuracy. The construction of the “TCM syndrome-comorbidity” prediction model may be very helpful in assisting the early diagnosis of diabetic comorbidity. However, due to the limitations of the data, only ten common comorbidities were included in this study. The data volume will be expanded in the future to further determine the relationship between type 2 diabetic comorbidities and different TCM syndromes.

## Figures and Tables

**Table 1:** General information of type 2 diabetes patients with comorbidity.

Variable	Value
Gender, *n* (%)	
Male	470 (47.24)
Female	525 (52.76)
Age (years), M (Q1, Q3)	56 (46, 65)
Age (years), *n* (%)	
18–39	130 (13.06)
40–59	465 (46.73)
60–79	374 (37.59)
≥80	26 (2.61)
Frequency of visit, M (Q1, Q3)	2 (1,4)
Frequency of visit, *n* (%)	
1–3	717 (73.31)
4–6	144 (14.72)
7–9	42 (4.29)
≥10	75 (7.67)
Comorbidity, *n* (%)	
1-2	3019 (86.82)
3-4	396 (11.39)
≥5	62 (1.78)
TCM syndrome, *n* (%)	
1-2	2562 (75.17)
3-4	829 (24.33)
≥5	16 (0.47)

M: median; Q1: lower quartile; Q3: upper quartile.

## Data Availability

The data used to support the findings of this study are included within the article.
